# A generic concept to overcome bandgap limitations for designing highly efficient multi-junction photovoltaic cells

**DOI:** 10.1038/ncomms8730

**Published:** 2015-07-16

**Authors:** Fei Guo, Ning Li, Frank W. Fecher, Nicola Gasparini, Cesar Omar Ramirez Quiroz, Carina Bronnbauer, Yi Hou, Vuk V. Radmilović, Velimir R. Radmilović, Erdmann Spiecker, Karen Forberich, Christoph J. Brabec

**Affiliations:** 1 Institute of Materials for Electronics and Energy Technology (i-MEET), Friedrich-Alexander University Erlangen-Nürnberg, Martensstrasse 7, 91058 Erlangen, Germany.; 2 Bavarian Center for Applied Energy Research (ZAE Bayern), Haberstrasse 2a, 91058 Erlangen, Germany.; 3 Erlangen Graduate School in Advanced Optical Technologies (SAOT), Friedrich-Alexander-University Erlangen-Nürnberg, Paul-Gordan-Str. 6, 91052 Erlangen, Germany.; 4 Center for Nanoanalysis and Electron Microscopy (CENEM), Friedrich-Alexander University Erlangen-Nürnberg, Cauerstrasse 6, 91058 Erlangen, Germany.; 5 Innovation Center, Faculty of Technology and Metallurgy, University of Belgrade, Karnegijeva 4, 11120 Belgrade, Serbia.; 6 Nanotechnology and Functional Materials Center, Faculty of Technology and Metallurgy, University of Belgrade, Karnegijeva 4, 11120 Belgrade, Serbia.

## Abstract

The multi-junction concept is the most relevant approach to overcome the Shockley–Queisser limit for single-junction photovoltaic cells. The record efficiencies of several types of solar technologies are held by series-connected tandem configurations. However, the stringent current-matching criterion presents primarily a material challenge and permanently requires developing and processing novel semiconductors with desired bandgaps and thicknesses. Here we report a generic concept to alleviate this limitation. By integrating series- and parallel-interconnections into a triple-junction configuration, we find significantly relaxed material selection and current-matching constraints. To illustrate the versatile applicability of the proposed triple-junction concept, organic and organic-inorganic hybrid triple-junction solar cells are constructed by printing methods. High fill factors up to 68% without resistive losses are achieved for both organic and hybrid triple-junction devices. Series/parallel triple-junction cells with organic, as well as perovskite-based subcells may become a key technology to further advance the efficiency roadmap of the existing photovoltaic technologies.

## Abstract (web summary)

The efficiency of a single-junction photovoltaic cell is constrained by the Shockley-Queisser limit. Here, the authors adopt a triple-junction configuration which relaxes material and current-matching constraints, providing a generic strategy for advancing the efficiency roadmap of photovoltaic technologies.

## Graphical abstract

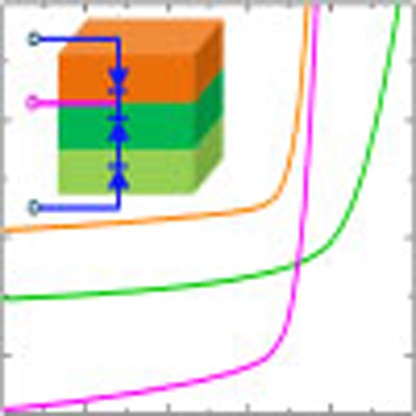

The power conversion efficiency (PCE) of a single-junction photovoltaic cell is fundamentally constrained by the Shockley–Queisser limit^1^. The record efficiencies of few solar technologies, such as single-crystal silicon, CuInGaSe_2_, CdTe and GaAs solar cells are constantly shrinking the gap to their fundamental efficiency limits^2^. To push the performances of these solar technologies beyond the Shockley–Queisser limit, several approaches have been proposed, for instance, up-conversion^3^, multi-junction configuration^4^,^5^,^6^, multiple exciton generation^7^,^8^ and concentrator cells, and so on. Among them, the multi-junction concept is one of the most promising candidates that allows to simultaneously address the two dominant loss mechanisms^4^, namely, sub-bandgap transmission and thermalization losses, which account for >55% of the total energy of the solar radiation^9^.

The conventional series-connected multi-junction cells are most successful in permanently enhancing the record efficiencies of the respective solar technologies^2^. However, one distinct drawback of the series-connected configuration is the stringent current-matching criterion, which requires careful bandgap engineering in combination with an excellent control of the thicknesses of the respective subcells. Therefore, many high-performance semiconductors with high external quantum efficiency (EQE) in the NIR absorption range exhibit limited applicability for multi-junction operation, as the perfectly matching semiconductor for the front or back subcells is missing. In contrast to the series-connection, a parallel-connection does not require current matching but instead voltage matching. The principle of voltage matching also constrains a semiconductor's applicability with respect to its bandgap, as well as inherently bears potential performance losses with respect to non-ideal open circuit voltages (*V*_OC_). However, the parallel-connection is more difficult to adapt and optimize for the high-performance semiconductors with non-tunable bandgaps, such as single-crystal silicon or CdTe.

In this manuscript, we present an interconnection approach as a technologically attractive solution to address all these challenges. We propose to deposit a transparent counter electrode and parallel-connect these ‘semitransparent' high-efficiency cells with one or more deep NIR sensitizers as back subcells. This is a feasible approach as there are indeed several types of far NIR semiconductors like organic donors^10^,^11^ and quantum dots^12^,^13^ with an extended absorption beyond 1,000 nm. The benefit of this series/parallel (SP) multi-junction design is based on the fact that—first, the absorber layer of the front ‘semitransparent' hero cell can be made arbitrarily thick (as there is no requirement for current matching), so that this subcell can achieve almost the same efficiency as the opaque single-junction reference. Second, the *V*_OC_ of the back cell, which is consisting of a series-connection of deep NIR absorbers, can be custom fabricated by stacking an arbitrary sequence of semiconductors with different bandgaps in series. This strategy dramatically reduces the material requirements for voltage matching when parallel-connected to the front subcell. As a consequence, the net photocurrent gain contributed by the deep NIR subcells ultimately adds up to the overall photocurrent of the multi-junction photovoltaic cell.

In the following, we start with the demonstration of the integrated SP triple-junction cells for solution-processed organic solar cells. Optical simulations are performed to predict the efficiency potential of different types of triple-junction configurations. Based on rational interface engineering, two fully solution-processed intermediate layers are successively developed, allowing effectively coupling the three cells into a SP interconnected triple-junction configuration. We then extend the concept to the recently emerging perovskite solar cells. A series-connected organic tandem solar cell absorbing photons in the NIR range is stacked in a four-terminal configuration behind a semitransparent perovskite cell. Due to the well-matched *V*_OC_ between the perovskite cell and the series-connected tandem cell, the photocurrent delivered by the organic tandem cell, up to 2 mA cm^−2^, directly contributes to the performance enhancement of the perovskite cell. Thus, the novel triple-junction concept demonstrated in this work provides an easy but elegant way to manufacture highly efficient photovoltaic cells, not only for conventional but also for the emerging solar technologies.

## Results

### Efficiency limits of triple-junction organic solar cells

**Figure 1:**
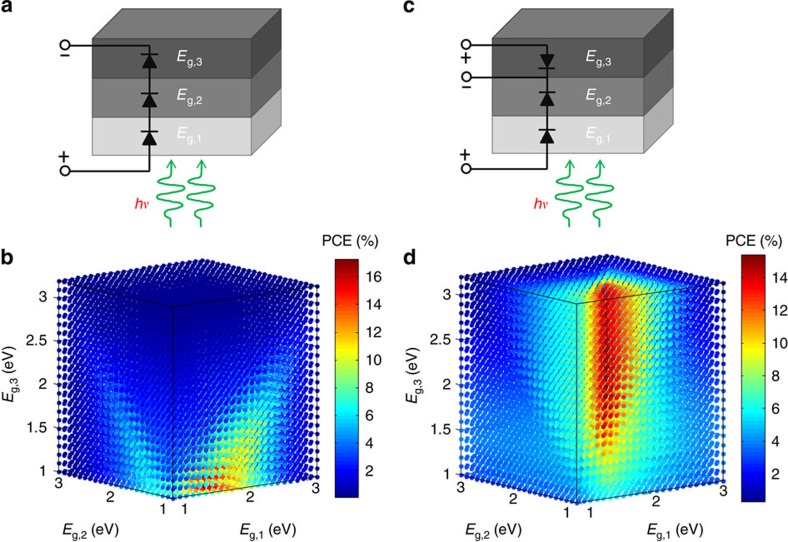
Comparison of efficiency limit for the two types of triple-junction organic solar cells. (**a**) Equivalent electronic circuit of the series/series (SS) triple-junction organic solar cells. (**b**) Three-dimensional efficiency map of the SS triple-junction devices as a function of the absorbers' bandgaps (*E*_g_) of the three subcells. (**c**) Equivalent electronic circuit of the series/parallel (SP) triple-junction devices. (**d**) Three-dimensional efficiency map of the SP triple-junction organic solar cells as a function of the absorbers' bandgaps of the three subcells.

There are in total four types of device configurations for a triple-junction solar cell, designated as series/series (SS, Fig. 1a), series/parallel (SP, Fig. 1c), parallel/series (PS, Supplementary Fig. 1a) and parallel/parallel (PP, Supplementary Fig. 1b). The SP and PS configurations are distinguished by the stacking sequence of the two interconnections (parallel and series) depending on which interconnection the light passes through first. For organic solar cells, we followed the model proposed by Dennler *et al*.^14^,^15^ to calculate the efficiency potential for the four types of triple-junction architectures as a function of the bandgaps of three absorbers. Detailed assumptions and calculation procedure are presented in the Supplementary Note 1. The outcome of the calculations showed that maximum efficiencies of 17.29%, 17.89%, 15.41% and 13.95% are achievable for SS, PS, SP and PP configurations, respectively. Comparing the four possible interconnections, although the SS and PS configurations demonstrate higher maximum efficiencies, it is apparent that the SP and PP interconnections could offer a wider range of material combinations to reach their highest efficiencies. From a practical point of view, however, the PP interconnection is too complex to process due to the necessity of introducing two transparent intermediate electrodes. Accordingly, the SP interconnection provides a more feasible approach to reach its theoretical efficiency limit. Hereafter, we shall experimentally show that the SP triple-junction configuration can be fabricated with the intermediate electrode and all the semiconducting layers solution-processed.

### Fabrication of bottom DPP–DPP subcell

**Figure 2:**
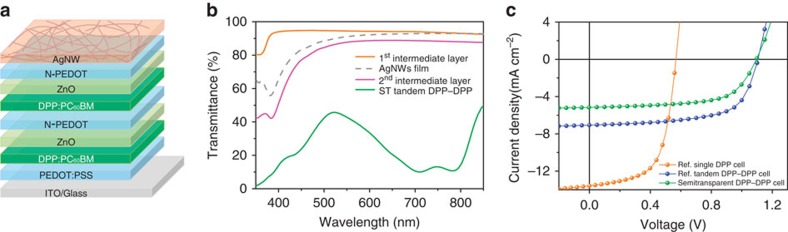
Semitransparent tandem device structure and performance. (**a**) Schematic architecture of the semitransparent series-tandem solar cells (DPP–DPP) with AgNWs top electrode. (**b**) Transmittance spectra of the two intermediate layers used in the SP triple-junction solar cells. (**c**) Typical *J*–*V* curves of the single-junction DPP reference cell, tandem DPP–DPP reference cell and the semitransparent tandem DPP–DPP cell with AgNW top electrode. The device structure of the single and tandem reference cells are: ‘Glass/ITO/PEDOT:PSS/DPP:PC_60_BM/Ca/Ag' and ‘Glass/ITO/PEDOT:PSS/DPP:PC_60_BM/ZnO/N-PEDOT/DPP:PC_60_BM/Ca/Ag'.

We began the fabrication of the SP triple-junction devices by designing and processing a semitransparent series-connected double-junction solar cell, as shown in Fig. 2a. We chose a diketopyrrolopyrrole-based low bandgap polymer pDPP5T-2 (abbreviated as DPP) blended with [6,6]-phenyl-C_61_-butyric acid methyl ester (PC_60_BM) as the photoactive layer of the two front subcells^16^,^17^, because the main absorption of this heterojunction extends to the near-infrared range with an absorption minimum between 450 and 650 nm (Supplementary Fig. 2). This absorption characteristic allows the transmitted photons to be absorbed by a wider bandgap top subcell. For series-connected tandem solar cells, the essential component is to construct an efficient intermediate layer serving as charge recombination zone for electrons and holes generated from subcells^6^,^18^,^19^,^20^,^21^,^22^,^23^,^24^,^25^. Herein, we chose ZnO and neutral PEDOT:PSS (N-PEDOT) as the N- and P-type charge extraction materials, respectively, because the work functions of the two materials match well with the energy levels of the donor DPP and acceptor PC_60_BM^20^,^23^. The transmittance spectrum of ZnO/N-PEDOT, the first intermediate layer, is depicted in Fig. 2b. The average transmittance of 94.2% in the range of 350–850 nm ensures minimal optical losses from these interface layers. To evaluate the as-designed recombination contacts, series-connected reference tandem cells using DPP:PC_60_BM as two identical active layers (denoted as DPP–DPP) were first constructed. As shown in Fig. 2c, the as-prepared opaque tandem device with evaporated Ca/Ag top electrode (15 nm/100 nm) shows a fill factor (FF) of 64.3% along with a *V*_OC_ of 1.1 V being the sum of two single-junction reference cells (Table 1). These results demonstrated the excellent functionality of the ZnO/N-PEDOT intermediate layer in the series-connected tandem architecture.

Now, the challenge remains to replace the vacuum-deposited metal electrode with a solution-processed, highly transparent electrode without deteriorating the performance of the established subcells beneath. We chose silver nanowires (AgNWs) as the intermediate electrode for our triple-junction devices because of their high transparency and low sheet resistance as well as the facile solution processability^26^,^27^,^28^,^29^,^30^. Our recent work demonstrated that a thin layer of ZnO nanoparticles can effectively conduct electrons to the AgNW electrode and, more importantly, enable the deposition of the AgNW electrode by doctor blading from water-based solution.^16^,^17^ However, both ZnO and AgNW layers are obviously not compact enough to protect the underlying subcells from solvent infiltration during the top subcell deposition. We have, therefore, additionally introduced a thin N-PEDOT layer between the ZnO and AgNWs to realize the second intermediate layer consisting of ‘ZnO/N-PEDOT/AgNWs' (second intermediate layer). Optical transmittance spectra of this intermediate layer and the entire semitransparent tandem DPP–DPP solar cell are shown in Fig. 2b. It is worth mentioning that our second intermediate layer with incorporated AgNWs exhibits an average transmittance of 84.5% (400–800 nm), which is a distinct advantage over evaporated thin metal films with low transmittance of ∼30–50% as middle electrode in realizing parallel-connection.^31^,^32^ Noticeably, the semitransparent tandem DPP–DPP cell shows an average transmittance of 35.6% in the range of 450–650 nm, which ensures for most wide bandgap materials to be applicable as top subcell to effectively harvest the transmitted photons.

A typical current density versus voltage (*J–V*) characteristic of the as-prepared semitransparent tandem solar cells (Fig. 2c) exhibits a *V*_OC_ of 1.10 V, which is identical to the reference tandem cell, suggesting the effective incorporation of AgNWs as the top electrode. Due to the lack of the back reflective electrode, the semitransparent tandem device shows a relatively low short circuit current (*J*_SC_) of 5.16 mA cm^−2^. In combination with the still high FF of 63.0%, these results provide sufficient evidence that the solution-deposited AgNW meshes are highly compatible with the underlying layers without compromising the device performance. In combination with our previous findings that the as-designed intermediate layer was able to resist high boiling-point solvent rinsing (chlorobenzene and dichlorobenzene)^16^, we expect that the successively established two intermediate layers are capable of coupling the series- and parallel-connected three cells into a monolithically deposited triple-junction stack.

### Top subcell fabrication

In our SP triple-junction devices, the top cell is connected in parallel with the bottom series-tandem cell which gives a *V*_OC_ of 1.1 V. To match the voltage between the parallel-connected components and thereby maximize the overall efficiency, a top cell with a *V*_OC_ value identical or close to the *V*_OC_ of the bottom series-tandem cell is desired. Here to demonstrate the general application of our SP triple-junction architecture, we studied two wide bandgap polymers, poly[N-9″-hepta-decanyl-2,7-carbazole-alt-5,5-(4′,7′-di-2-thienyl-2′,1′,3′-benzothiadiazole)] (PCDTBT, *E*_g_, 1.87 eV) and OPV12 (*E*_g_, 1.73 eV)^33^, as the top subcells, which give *V*_OC_ values of ∼0.9 V and 0.8 V when mixed with phenyl-C_71_-butyric acid methyl ester (PC_70_BM) and PC_60_BM, respectively. In addition, as indicated in Supplementary Fig. 2, the absorption profiles of the two active layers are complementary with that of DPP:PC_60_BM, suggesting they are appropriate material combinations for manufacturing multi-junction devices.

**Figure 3:**
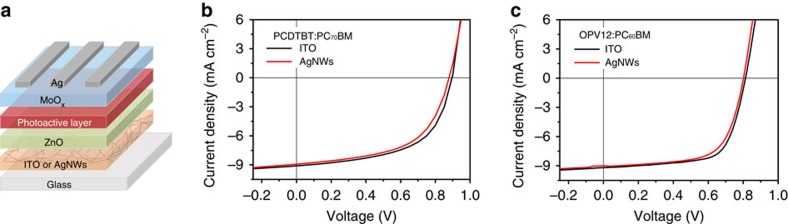
Device structure and photovoltaic performance of single-junction cells. (**a**) Device architecture of inverted solar cells with AgNW bottom electrode. (**b**,**c**) Typical *J–V* curves of single-junction reference cells of PCDTBT:PC_70_BM (**b**) and OPV12:PC_60_BM (**c**) deposited on ITO and AgNWs-coated glass substrates.

To verify the compatibility of the two wide bandgap donors with the AgNW electrode, single-junction reference cells of PCDTBT:PC_70_BM and OPV12:PC_60_BM were first processed on both indium tin oxide (ITO) and AgNWs-coated glass substrates for comparison (Fig. 3a). Typical *J–V* characteristics of the as-prepared single-junction devices are displayed in Fig. 3b,c and the key photovoltaic parameters are summarized in Table 1. Comparable device performances in terms of *V*_OC_, *J*_SC_ and PCE were observed for the two photoactive blends independent of bottom electrode. The slightly lower FFs for the devices fabricated on AgNWs as compared with the ITO counterparts can be ascribed to the higher series resistance (*R*_S_), probably resulting from the contact resistance between the AgNWs and ZnO. Nevertheless, these results suggest the excellent optoelectronic properties of the AgNWs that are compatible with different polymer donors. Indeed, independent measurement of the AgNW electrode employed in the current study shows an average visible transmittance of ∼90% (Fig. 2b) and a sheet resistance of ∼10 Ω sq^−1^, which is comparable to commonly used ITO electrodes.

### Organic triple-junction solar cells

**Figure 4:**
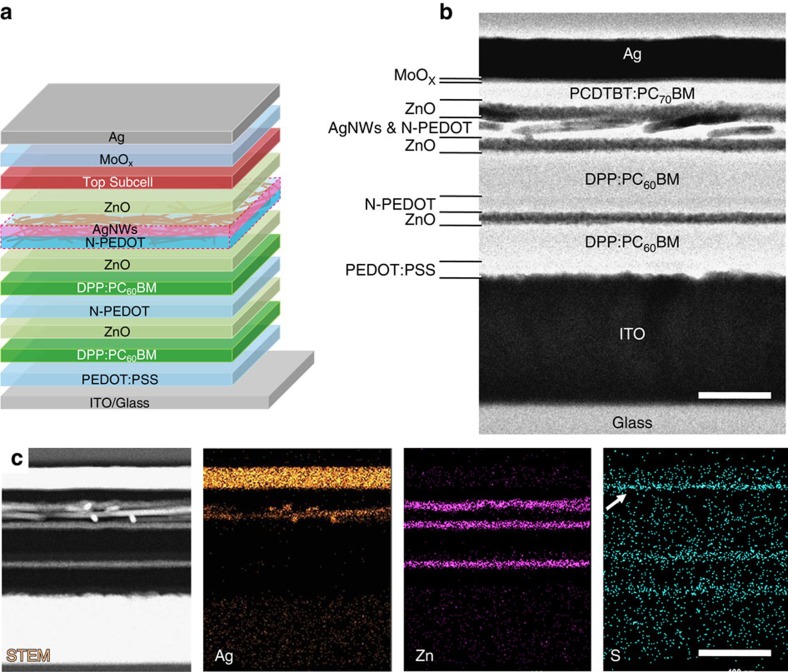
Microstructure of the triple-junction solar cell. (**a**) Device architecture of the SP triple-junction solar cell. (**b**) A cross-sectional TEM image of the as-prepared triple-junction solar cell. The scale bar, 200 nm. (**c**) STEM image of the cross-section and EDS elemental (Ag, Zn, S) maps. Note that the strongest top band (indicated by arrow) in the sulphur map belongs to molybdenum because of overlapping of S-K^α^ (2.307 keV) and Mo-L^α^ (2.293 keV) lines. The scale bar, 400 nm.

Having successfully constructed the individual bottom semitransparent tandem subcells and top subcell, in combination with the verified robust intermediate layers we now complete the fabrication of the entire SP triple-junction solar cells. Figure 4a shows the schematic illustration of the SP triple-junction cell design, where the bottom series-connected tandem subcells in a normal structure are electrically connected in parallel with the top inverted subcell. The middle AgNW layer in this triple-junction device serves as a common cathode to collect electrons created by the subcells. It is worth mentioning that we have employed a simple modified doctor blading technique to coat the AgNW electrode^16^, which enables the deposition of the NW film in a stripe and thereby eliminates any subsequent patterning steps. Detailed description of the device fabrication procedure is presented in the Methods section and schematically illustrated in Supplementary Fig. 3.

A cross-sectional transmission electron microscopy (TEM) image of a SP triple-junction solar cell is shown in Fig. 4b. The liftout sample was prepared using a focused ion beam (FIB, FEI Helios NanoLab 660) and imaged subsequently with the TITAN^3^ aberration-corrected TEM. All the individual layers of the solar cell can be clearly distinguished in the scanning TEM (STEM) image without any physical damage. It should be noted that, even though interlayer mixing between the AgNWs and the underlying N-PEDOT layer is observed, it does not negatively affect the device performance since the N-PEDOT in the stack purely acts as a ‘solvent protection' layer. On contrary, the fact that the AgNWs partially sink into N-PEDOT can reduce the roughness of the NW networks, which is beneficial for building the upper few layers and further reduces the possibility of shunts in the top subcell. The STEM energy dispersive X-ray spectrometry (EDS) elemental maps (Ag, Zn and S) of the cross-section shown in Fig. 4c confirms a well-organized layer stack.

**Figure 5:**
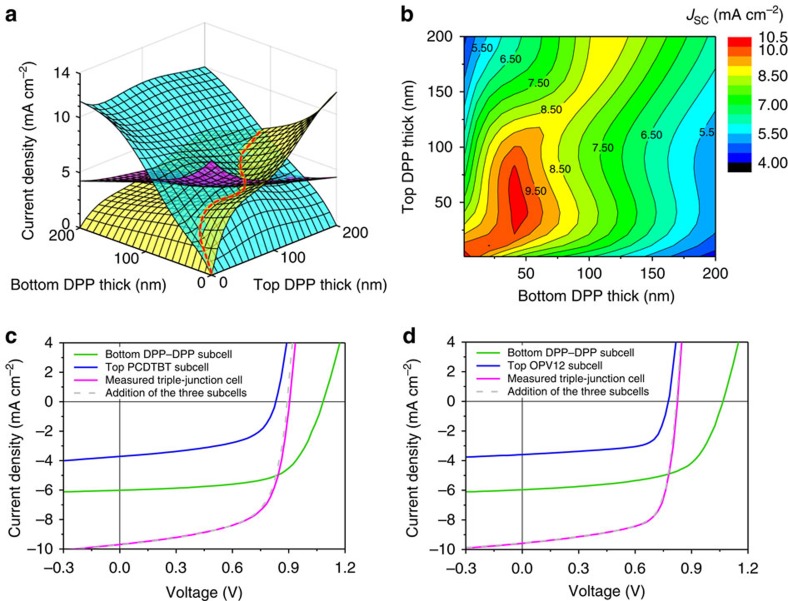
Optical simulations and photovoltaic characteristics of the SP triple-junction solar cells. (**a**) Simulated current density distribution of the three subcells as a function of the thicknesses of bottom two DPP:PC_60_BM layers. (**b**) Contour plot of current density distribution of the entire triple-junction devices (DPP–DPP/PCDTBT) as a function of the thicknesses of bottom DPP:PC_60_BM layers. Note that in these two simulations the top PCDTBT:PC_70_BM layer thickness is fixed to 80 nm, corresponding to the optimized thickness in their single-junction state. (**c**,**d**) *J–V* characteristics of the investigated triple-junction cells and the constituent bottom series-tandem subcells and top subcell, (**c**) DPP–DPP/PCDTBT, (**d**) DPP–DPP/OPV12. The light grey dashed lines indicate the numerical addition of the bottom series-tandem subcells and the top subcell.

Simultaneously, optical simulations based on the transfer matrix formalism were carried out to calculate the current generation in the individual subcells^34^,^35^, which can provide valuable guidance for optimization of our SP triple-junction devices. Taking advantage of the fact that parallel-connection does not require current matching, and therefore balancing the current flow in the bottom series-tandem DPP–DPP cells is of critical significance. In the case of DPP–DPP/PCDTBT triple-junction devices, for the purpose of simplicity we fixed the thickness of the top PCDTBT:PC_70_BM to be 80 nm corresponding to the thickness of optimized single-junction reference cells. Detailed assumption and calculation procedure are presented in the Supplementary Note 2. The outcome of the simulations is shown in Fig. 5a, illustrating the interplay of the photocurrent generation in the three subcells. It is obvious that to maximize the use of incident photons, the thicknesses of the two DPP:PC_60_BM active layers should follow the red dashed line where the photocurrents generated in the two subcells are identical. Taking the photocurrent of the top subcell PCDTBT:PC_70_BM into consideration, the resulting contour plot of the current density distribution of the entire triple-junction solar cells as a function of the thicknesses of two DPP:PC_60_BM layers is depicted in Fig. 5b. One can see that maximum photocurrents of ∼10 mA cm^−2^ are achievable for our DPP–DPP/PCDTBT triple-junction devices when the thicknesses of the bottom and top DPP:PC_60_BM subcells are in the range of 30–60 nm and 35–80 nm, respectively. Similar simulation results for the triple-junction DPP–DPP/OPV12 devices are presented in Supplementary Fig. 4.

Experimentally, to evaluate the photovoltaic performances of the subcells, we designed a three-terminal layout to prepare our SP triple-junction solar cells, which allows us to detect the *J–V* characteristics of both the bottom series-tandem subcell and the top subcell within their connected state (Supplementary Fig. 3). Figure 5c,d show the typical *J–V* curves of the constructed triple-junction solar cells, DPP–DPP/PCDTBT and DPP–DPP/OPV12, along with the constituent subcells, respectively. The key photovoltaic parameters are listed in Table 2. It can be seen that the two triple-junction cells achieved *J*_SC_ of 9.67 mA cm^−2^ (DPP–DPP/PCDTBT) and 9.55 mA cm^−2^ (DPP–DPP/OPV12) which is in good agreement with the optical simulations. Noticeably, from Table 2 we can see that the measured photocurrents of the triple-junction cells are more or less identical to the sum *J*_SC_ values extracted from the respective bottom DPP–DPP subcells and top PCDTBT or OPV12 subcells. The *J*_SC_ values of the top subcells were verified with EQE measurement (Supplementary Fig. 5) and the values calculated by integrating the EQE curve with standard AM1.5 G spectrum show a good agreement with the measured *J*_SC_ values. Moreover, as depicted in Fig. 5c,d, if we mathematically add the *J–V* curves of the DPP–DPP subcells with the top PCDTBT or OPV12 subcell at each voltage bias (*V*_bias_), a perfect fitting of the constructed *J–V* curve with the experimentally measured *J–V* curve of the triple-junction device is observed, which is consistent with Kirchhoff's law. These observations provide sufficient evidence that there are no resistive losses for the intermediate AgNW electrode in terms of collecting charge carriers.

For both triple-junction solar cells, the bottom series-connected DPP–DPP subcells showed *V*_OC_ values of 1.07–1.08 V, indicating that the solution-processing of the upper layers imposes no negative effect on the established bottom subcells. Compared with the reference DPP–DPP tandem cell, the slightly reduced *V*_OC_ of 0.02–0.03 V can be attributed to shadow effect^36^, because a mask with an aperture smaller than either electrode was adopted to define the active area during the *J–V* measurement. While the reduced light intensity filtered by the front DPP–DPP subcells further slightly decreased the *V*_OC_ of the back PCDTBT:PC_70_BM or OPV12:PC_60_BM subcells by a value of 0.03–0.05 V.

For solar cells with ideal diode characteristics, the *V*_OC_ of the parallel-connected tandem cells would be strictly restricted by the subcell, which delivers low *V*_OC_. In real parallel-connected solar cells, however, the *V*_OC_ of the tandem cells can be close either to the subcell with high *V*_OC_ or to the subcell with low *V*_OC_ depending on the series resistance of the subcells^37^. In our parallel-connected constituent subcells, the two top subcells showed series resistance of ∼1 Ω cm^2^ which is almost eight times lower than those of bottom DPP–DPP subcells (Table 2). This is due to the fact that the charge injections in the top subcells are higher than in the bottom subcells at *V*_bias_>*V*_OC_. Consequently, the top subcells showed steeper slopes at *V*_bias_>*V*_OC_ compared with the bottom subcells. Taking Kirchhoff's law into consideration, these circumstances lead to the *V*_OC_ values of our triple-junction cells close to the top subcells which exhibited lower *V*_OC_.

Moreover, it should be noted that although our triple-junction cells have achieved PCEs of 5.35 and 5.43%, which are higher than either one of the single-junction reference devices, those values are still ∼0.4% lower than the sum PCEs of the incorporated subcells. These PCE losses are mainly attributed to the relatively low *V*_OC_ of triple-junction that is close to the top subcells, and this suppression can be readily eliminated by employing high-performance top subcells with *V*_OC_ matched to the bottom series-connected subcells. Nevertheless, these results in combination with the high FFs of up to 68% eventually suggest that the engineered intermediate layers have efficiently coupled the three cells into triple-junction with an integrated SP interconnection.

### Hybrid triple-junction solar cells

Organometal halide perovskites have emerged as promising materials that enable fabrication of highly efficient solar cells by solution deposition^38^,^39^,^40^. Although efficiencies exceeding 15% have been frequently reported, it is widely acknowledged that the moderate bandgap of 1.55 eV offers enormous potential to further enhance the device efficiency by using multi-junction configurations^39^,^40^. Given that the perovskite single cell (mixed halide CH_3_NH_3_PbI_3−*x*_Cl_*x*_) provides a high *V*_OC_ of ∼1 V, which is comparable to our series-connected DPP–DPP cells, it is straightforward to fabricate a PS connected triple-junction device by placing a DPP–DPP cell behind a semitransparent perovskite cell, and thereby adding up the total current density for the hybrid triple-junction device.

**Figure 6:**
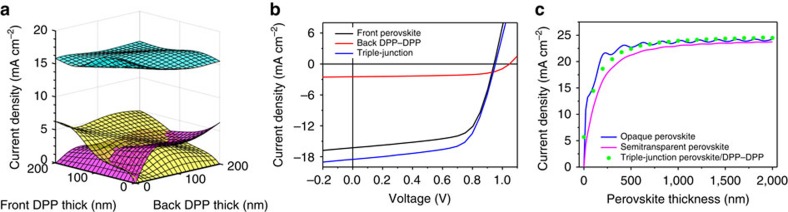
Simulation and experimental demonstration of hybrid triple-junction solar cells with a parallel/series interconnection. (**a**) Calculated *J*_SC_ distribution of the three subcells as a function of the back two DPP:PC_60_BM film thicknesses. The thickness of the front perovskite layer is fixed to 200 nm which corresponds to the thickness of the optimized reference cells. (**b**) Measured *J–V* curves of the two constituent subcells and the triple-connected device. (**c**) Calculated *J*_SC_ values of the semitransparent, opaque perovskite cells and the proposed triple-junction devices (perovskite/DPP–DPP) as a function of layer thickness of the perovskite.

Figure 6a shows the calculated *J*_SC_ distribution of the three subcells of the hybrid triple-junction device as a function of the thicknesses of the back two DPP cells. We used an internal quantum efficiency of 100% for our simulation^41^. The front 200-nm-thick perovskite cell exhibits a *J*_SC_ of ∼16 mA cm^−2^, which is slightly affected by the interference of the device. The EQE measurement of a prepared semitransparent perovskite cell (Supplementary Fig. 6) gives a current density of 15.98 mA cm^−2^ which is in good agreement with the simulation values (Supplementary Methods for fabrication details). A current density of up to 3 mA cm^−2^ is calculated for the series-connected DPP–DPP tandem cell, as a benefit of the average 53.4% transmittance (650 and 850 nm) of the semitransparent perovksite cell (Supplementary Fig. 7). Figure 6b shows the measured *J–V* curves of the experimentally constructed hybrid triple-junction solar cell and the corresponding subcells. The semitransparent perovskite device shows a *J*_SC_=16.28 mA cm^−2^, *V*_OC_=0.94 V and FF=65.6%, yielding a PCE of 10.04%. The hybrid triple-junction device perovskite/DPP–DPP exhibits a high current density of 18.51 mA cm^−2^ with about 2 mA cm^−2^ contributed from the back DPP–DPP subcells. Together with the high FF of 64.5% and *V*_OC_ of 0.95 V, the hybrid triple-junction device shows a PCE value of 11.34%, corresponding to a PCE enhancement by 12.5%.

To illustrate the benefit of the hybrid triple-junction device, we further theoretically compared the current generation between the single opaque perovskite cells and the hybrid triple-junction devices using the same material combinations. As presented in Fig. 6c, the *J*_SC_ value of the triple-junction device reaches to the *J*_SC_ value of the opaque single-junction perovskite cell, for perovskite cells with a layer thickness of >300 nm. It should be noted that the absorption of the DPP polymer donor shows a red-shift of only ∼50 nm compared with the perovskite and, therefore, we expect a significant enhancement when deeper NIR sensitizers are used as back series-connected tandem cells.

## Discussion

We have experimentally demonstrated in this work, for the first time, solution-processed organic and hybrid triple-junction solar cells with integrated series- and parallel-interconnection. The optical simulations reveal that the as-proposed SP triple-junction organic solar cells hold the potential to achieve high efficiencies close to those of the fully series-connected counterparts, but allowing a much wider choice of material combinations. Through a rational interface layer design, triple-junction devices with all solution-processed intermediate layers achieved PCEs of ∼5.4% with FFs of up to 68%. Currently, the efficiency of our SP triple-junction devices is mainly limited by the mismatch of the *V*_OC_ of the top subcell with the *V*_OC_ of the bottom series-connected tandem subcells. Based on the convenient solution-processing along with the impressive high FFs, we expect that significant enhancement in efficiency can be achieved by exploiting high-performance wide bandgap materials with matched *V*_OC_ in the back subcell. Alternatively, our results predict a significantly growing interest in ultra-low bandgap semiconductors allowing for more efficient light-harvesting for these SP triple-junction solar cells.

The general applicability of the proposed triple-junction configurations has also been verified in organic-inorganic hybrid triple-junction devices. By combining a semitransparent perovskite cell with series-connected DPP–DPP cells in parallel, the fabricated hybrid triple-junction devices showed an efficiency improvement by 12.5% compared with the corresponding reference cells. Further, we believe that the novel, but generic, concept demonstrated in this work potentially provides a promising avenue to approach or exceed the Shockley–Queisser limit of many of the currently available high-performance semiconductors such as crystalline silicon, CdTe and perovskite solar cells^42^,^43^,^44^.

## Methods

### Materials

ITO-coated glass substrates (2.5 × 2.5) cm^2^ with a sheet resistance of 15 Ω sq^−1^ were purchased from Weidner Glas and patterned with laser before use. Light absorbers DPP, OPV12 and PCDTBT were purchased from BASF, Polyera and 1-Materials, respectively. PEDOT:PSS (Clevios, P VP AI 4083) and N-PEDOT (NT5-3417286/2) were obtained from Heraeus and Agfa, respectively. ZnO nanoparticles dispersed in isopropanol (Product N-10) and AgNW dispersion (ClearOhm Ink) were supplied by Nanograde AG and Cambrios Technologies Corporation, respectively. PC_60_BM (99.5%) and PC_70_BM (99%) were purchased from Solenne BV. All the materials were used as received without further purification.

### Fabrication of triple-junction devices

For our SP triple-junction organic solar cells, with the exception of bottom ITO-coated glass substrate and top evaporated MoO_*X*_/Ag electrode, all the layers were sequentially deposited using a doctor blade in ambient atmosphere. Prior to device fabrication, the laser-patterned ITO substrates were cleaned by ultra-sonication in acetone and isopropanol for 10 min each.

On the cleaned substrates, PEDOT:PSS (Clevious P VP Al 4083, 1:3 vol.% diluted in isopropanol) was firstly bladed and annealed at 140 °C for 5 min to obtain a layer thickness of ∼40 nm. On top of the dried PEDOT:PSS, the first photoactive layer consisting of DPP and PC_60_BM (1:2 wt.% dissolved in a mixed solvent of chloroform and o-dichlorobenzene (9:1 vol.%)) was deposited at 45 °C to obtain a thickness of ∼50 nm. The first intermediate layers, ZnO and N-PEDOT:PSS, were sequentially bladed at 50 °C and annealed at 80 °C for 5 min in air and the obtained layer thickness for both layers is ∼35 nm. The second active layer DPP:PC_60_BM with thickness of ∼80 nm was then coated on top of N-PEDOT at 55 °C. Afterwards, ZnO and N-PEDOT were again deposited onto the second DPP:PC_60_BM layer using the same coating parameters as for the first deposition. To deposit the intermediate electrode, ∼80-nm-thick AgNWs was bladed onto N-PEDOT at 45 °C and the resulting NW film showed a sheet resistance of ∼8 Ω sq^−1^. Successively, an electron extraction layer of ZnO was deposited on top of AgNWs using the same parameters, followed by blading the third active blend of PCDTBT:PC_70_BM at 60 °C. After all the solution-processed layers were completed, Q-tips dipped with toluene were used to clean the edges of the substrate to expose the bottom ITO and middle AgNW contacts. Finally, to complete the device fabrication, a 15-nm-thick MoO_*X*_ and 100-nm-thick Ag were thermally evaporated on top of PCDTBT:PC_70_BM through a shadow mask with an opening of 10.4 mm^2^.

Triple-junction solar cells DPP–DPP/OPV12 were prepared with the same processing procedure as device DPP–DPP/PCDTBT. To achieve a reliable contact between the middle AgNW electrode and probes of the measurement set-ups (*J–V* and EQE measurements), silver paste or evaporated silver was applied to the exposed AgNWs (Supplementary Fig. 3). Semitransparent DPP–DPP reference tandem cells with top AgNW electrode and the single-junction reference devices (PCDTBT:PC_70_BM and OPV12:PC_60_BM) with bottom AgNW electrode were fabricated using the same procedure as these subcells in the SP triple-junction cells.

The semitransparent perovskite (mixed halide CH_3_NH_3_PbI_3−*x*_Cl_*x*_) solar cells with a device structure of ITO/PEDOT:PSS/Perovskite/PC_60_BM/ZnO/AgNWs (Supplementary Fig. 5a) was fabricated using a procedure as described in the Supplementary Methods^45^. The hybrid triple-junction solar cell was assembled by stacking a series-connected opaque DPP–DPP as back subcell with a semitransparent perovskite device as front subcell. The parallel-connection between the semitransparent perovskite and series-connected DPP–DPP subcells was realized by external coupling using Ag paste.

### Characterization

Transmittance spectra of the intermediate layers and semitransparent devices were measured using a UV–vis-NIR spectrometer (Lambda 950, from Perkin Elmer). *J–V* curves of all the devices were recorded using a source measurement unit from BoTest. Illumination was provided by a solar simulator (Oriel Sol 1 A from Newport) with AM1.5G spectrum and light intensity of 100 mW cm^−2^, which was calibrated by a certified silicon solar cell. To guarantee the incident light to be able to illuminate on all the three electrodes with an overlapped active area, during the *J–V* measurement a mask with an aperture of 4.5 mm^2^ was used to define the cell area. The EQE spectra were recorded with an EQE measurement system (QE-R) from Enli Technology (Taiwan). The light intensity at each wavelength was calibrated with a standard single-crystal Si solar cell. Liftout sample for TEM was prepared with FEI Helios Nanolab 660 DualBeam FIB, from the area-of-interest containing all layers of the solar cell. A lamella containing a cross-section of the solar cell was then attached to a TEM half grid for final thinning. The final thickness of the liftout sample was kept <100 nm, to enable high quality conventional transmission electron microscopy (CTEM) imaging at an acceleration voltage of 200 kV. TEM was performed on the FEI TITAN^3^ Themis 60–300 double aberration-corrected microscope at the Center for Nanoanalysis and Electron Microscopy (CENEM), the University of Erlangen, equipped with the super-X energy dispersive spectrometer.

## Additional information

**How to cite this article:** Guo, F. *et al*. A generic concept to overcome bandgap limitations for designing highly efficient multi-junction photovoltaic cells. *Nat. Commun.* 6:7730 doi: 10.1038/ncomms8730 (2015).

## Supplementary Material

Supplementary Information Supplementary Figures 1-7, Supplementary Notes 1-2, Supplementary Methods and Supplementary References

## Figures and Tables

**Table 1:** Photovoltaic parameters of the single-junction devices (DPP, PCDTBT and OPV12) and the tandem DPP–DPP solar cells.

Device	Electrode bottom/top	*V*_OC_ (V)	FF (%)	*J*_SC_ (mA cm^−2^)	PCE (%)
DPP:PC_60_BM	ITO/Ag	0.56	62.1	13.5	4.69
Tandem	ITO/Ag	1.10	64.5	7.06	5.01
DPP–DPP cells	ITO/AgNWs	1.10	63.0	5.16	3.58
PCDTBT:PC_70_BM	ITO/Ag	0.89	57.1	9.03	4.59
	AgNWs/Ag	0.88	55.5	8.93	4.36
OPV12:PC_60_BM	ITO/Ag	0.81	67.8	9.12	5.01
	AgNWs/Ag	0.80	66.4	9.04	4.80

AgNW, silver nanowire; DPP, pDPP5T-2; PCE, power conversion efficiency.

**Table 2:** Photovoltaic parameters of the triple-junction cells and the constituent subcells.

Material combination	Devices	*V*_OC_ (V)	FF (%)	*J*_SC_ (mA cm^−2^)	PCE (%)	*R*_S_ (Ω cm^2^)
DPP–DPP/PCDTBT	Bottom DPP–DPP subcell	1.08	64.2	6.00	4.16	8.03
	Top PCDTBT subcell	0.82	54.9	3.70	1.66	1.15
	Triple connection	0.89	63.1	9.67	5.43	1.04
DPP–DPP/OPV12	Bottom DPP–DPP subcell	1.07	60.7	5.95	3.86	7.14
	Top OPV12 subcell	0.77	67.8	3.58	1.87	0.82
	Triple connection	0.82	68.4	9.55	5.35	0.71

DPP, pDPP5T-2; PCE, power conversion efficiency.

*R*_S_ of the cells were calculated at ∼2 V of the *J–V* curves measured in dark.
