# Supplementary material for: A generic concept to overcome bandgap limitations for designing highly efficient multi-junction photovoltaic cells
Source: Nat Commun. 2015 Jul 16;6:7730. doi: 10.1038/ncomms8730 (PMC4518253; doi:10.1038/ncomms8730)
Supplement: Supplementary Information — Supplementary Figures 1-7, Supplementary Notes 1-2, Supplementary Methods and Supplementary References [file ncomms8730-s1.pdf]

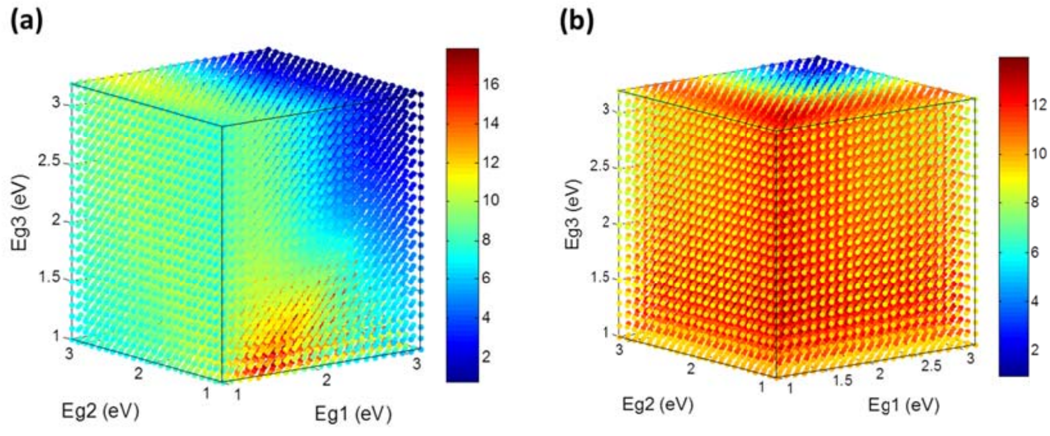

Supplementary Figure 1 | 3-Dimensional efficiency map of the PS (**a**) and PP (**b**) triple-junction organic solar cells as a function of the absorbers' band-gaps of the three sub-cells.

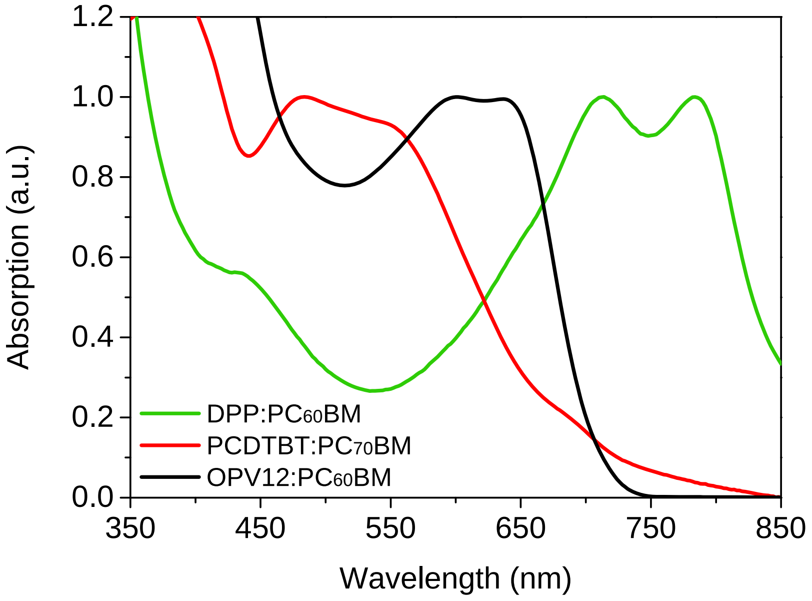

Supplementary Figure 2 | Absorption spectra of DPP:$PC_{60}BM$, PCDTBT:$PC_{70}BM$ and OPV12:$PC_{60}BM$ in solid film. The curves are normalized at the characteristic absorption peaks of the films.

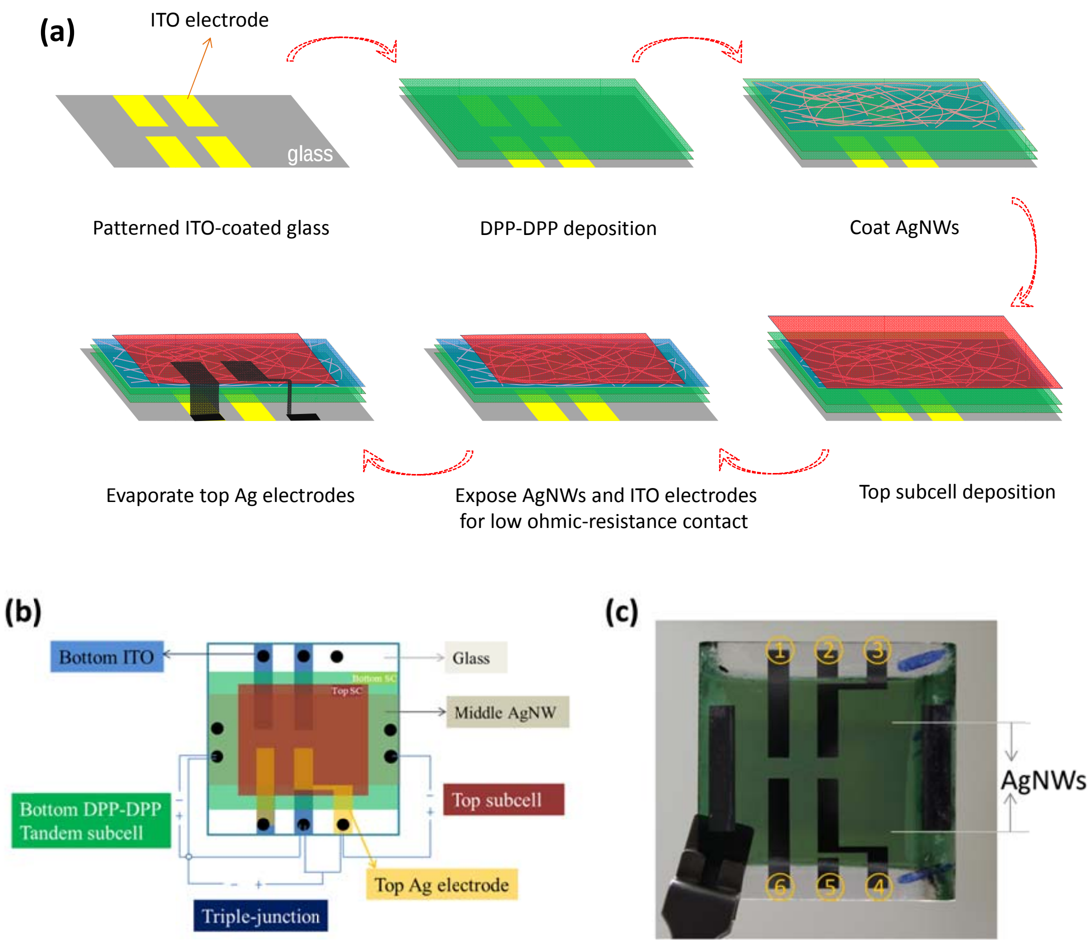

Supplementary Figure 3 | (**a**) Schematic illustration of the device fabrication from substrates preparation to the top electrode deposition. The deposition of intermediate AgNWs using a modified doctor blade can refer to our recent work.[1] (**b**) Schematic illustration of the three-terminal layout design (top view). In this device layout, two 2-terminal and two 3-terminal triple-junction cells are presented. (**c**) A digital photo of an as-fabricated triple-junction cell, where ① and ⑥ indicate two 2-terminal triple-junction cells. The other two devices are 3-terminal triple-junction cells, where the bottom DPP-DPP subcells can be accessed through ② and ⑤, while the top PCDTBT:$PC_{70}BM$ or OPV12:$PC_{60}BM$ subcells can be accessed via ③ and ④. In addition, the integrated AgNWs layer can be seen in the triple-junction solar cell.

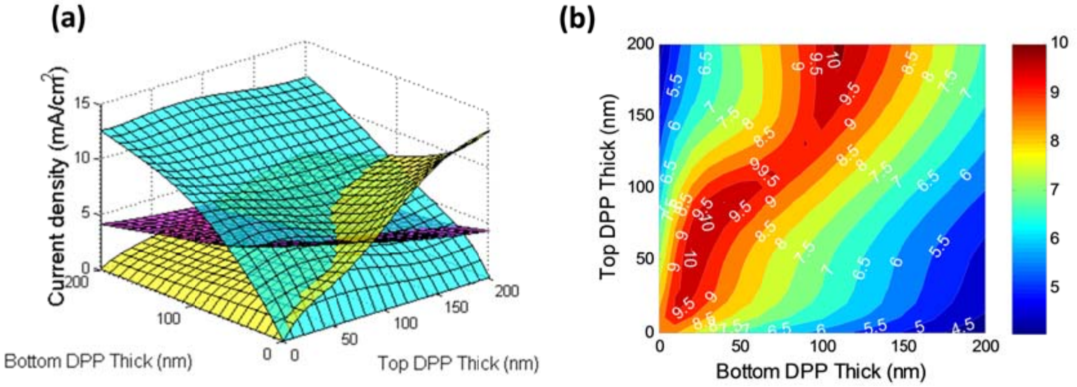

Supplementary Figure 4 | (**a**) Simulated current density distribution of the three subcells as a function of the thicknesses of bottom two DPP:PC_{60}BM layers. (**b**) Contour plot of current density distribution of the entire triple-junction devices (DPP-DPP/OPV12) as a function of the thicknesses of bottom DPP:PC_{60}BM layers. Note that in these two simulations the top OPV12:PC_{60}BM layer thickness is fixed to 200 nm, corresponding to the optimized thickness in their single-junction state.

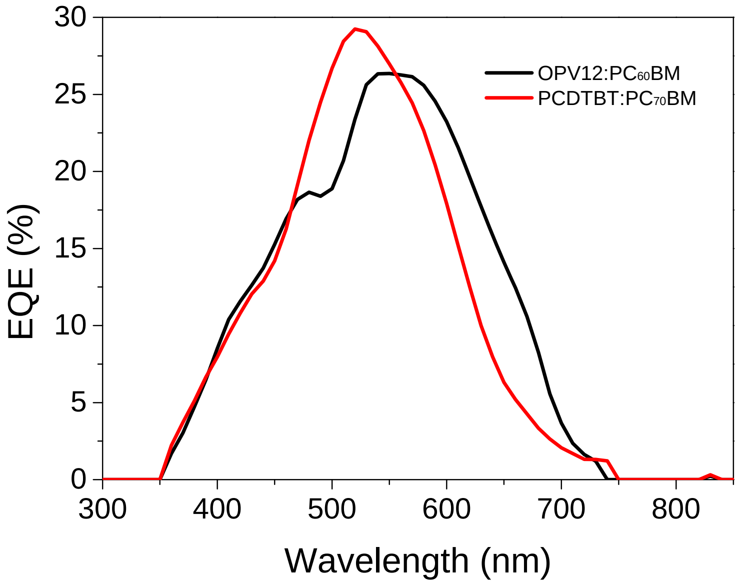

Supplementary Figure 5 | EQE characteristics of the two back subcells (OPV12:$PC_{60}BM$ and PCDTBT:$PC_{70}BM$) of the corresponding triple-junction organic solar cells. The integration of the two EQE curves with standard AM 1.5 G give current densities of 3.55 and 3.34 mA/cm^2 for the OPV12:$PC_{60}BM$ and PCDTBT:$PC_{70}BM$ subcell, respectively.

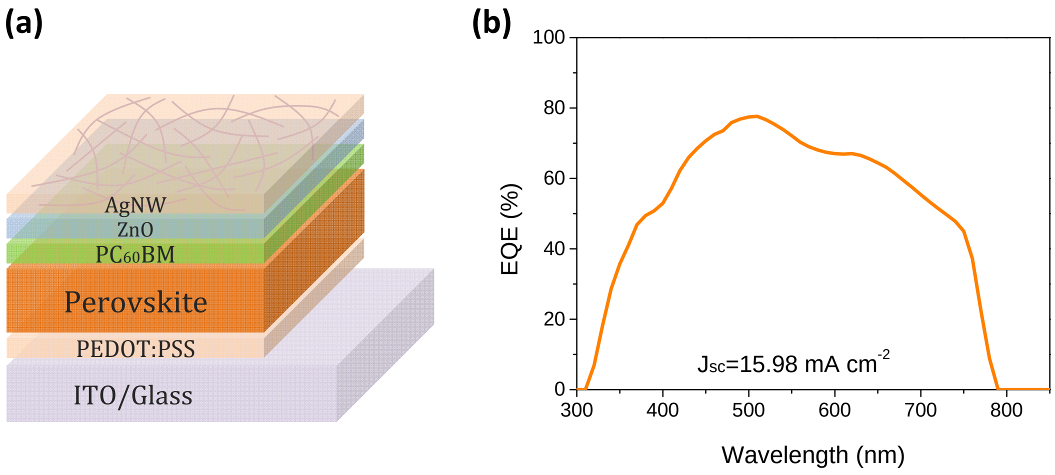

Supplementary Figure 6 │ (**a**) Architecture of the semitransparent perovskite solar cells. (**b**) A typical EQE characteristic of the prepared semitransparent perovskite devices which gives a J_{sc} value of 15.98 mA cm^{-2} by integrating the EQE curve with the standard AM 1.5G spectrum with intenstiy of 100 mW cm^{-2}.

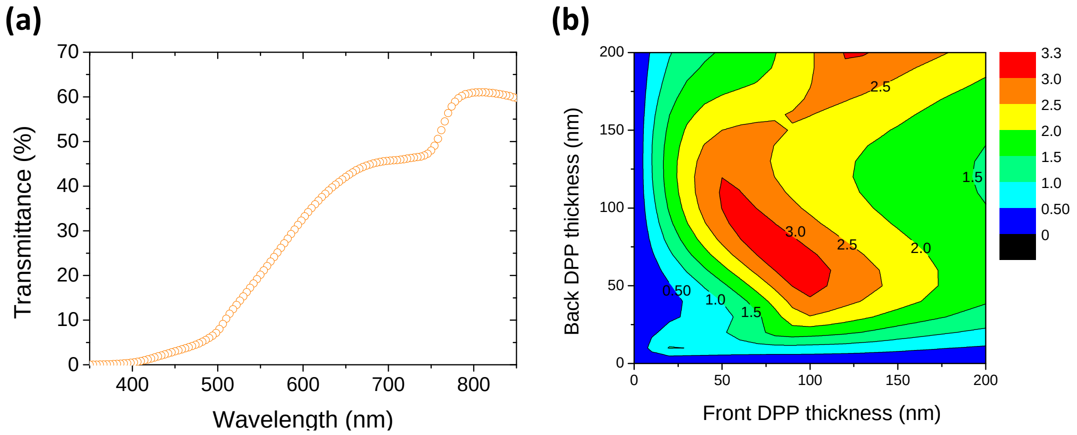

Supplementary Figure 7 │ (**a**) Transmittance spectrum of a semitransparent perovskite solar cell. (**b**) Current density distribution of the back series-connected DPP-DPP subcells. For the each layer thickness combination, we assume the J_{sc} of the tandem device equals to the subcell which delivers low J_{sc} values.

Supplementary Note 1

Efficiency calculation based on semi-empirical model

To calculate the efficiency limits of the four types of triple-junction organic photovoltaic (OPV) cells, we followed the models proposed by Scharber et al. and Dennler et al.,[2, 3] which had been used to calculate the limiting efficiencies of single-junction as well as series-connected double-junction OPV devices.

Basic assumptions:

EQE=0.65 (single junction cell with a reflective back electrode)

IQE=0.85 (both EQE and IQE are supposed to be constant in the whole absorption range)

FF = 0.65,

$V_{oc} = (E_g\text{-}0.6\text{ eV})/e$,[4] (1)

LossMirror = 0.15 (accounts for the absorption losses of the front subcell in the absence of a back reflective electrode)

Taking the mirror losses and absorption of the front subcells into consideration, the EQEs of the three subcells have to be modified depending upon their overlapping status. Generally, the EQE of the front subcell (EQE_1), middle subcell (EQE_2) and rear subcell (EQE_3) can be expressed as follows:

EQE_1 = EQE*(1-LossMirror); (2)

EQE_2 = (1-EQE_1/IQE)*EQE*(1-LossMirror); (3)

EQE_3 = (1-(EQE_1/IQE)-EQE_2/IQE)*EQE; (4)

$$J_{sc} = \frac{q}{hc}\int_{\lambda 1}^{\lambda 2} P_{AM1.5G}(\lambda)\times\lambda\times EQE(\lambda)d\lambda \quad (5)$$

According to Kirchoff's circuit law, the V_{oc} and J_{sc} of the series- or parallel-connected cells and their component subcells should follow these relationships.

For series connection

$$V_{oc,\tan} = V_{oc,1} + V_{oc,2} \quad (6)$$

$$J_{sc,\tan} = \min[J_{sc,1}; J_{sc,2}] \quad (7)$$

For parallel connection

$$V_{oc,\tan} = \min[V_{oc,1}; V_{oc,2}] \quad (8)$$

$$J_{sc,\tan} = J_{sc,1} + J_{sc,2} \quad (9)$$

Supplementary Note 2

Optical Simulations

Optical simulations were performed by means of Transfer Matrix Formalism.[5, 6] This method is widely adopted for calculation of transmittance, reflectance, absorbance and electric field distribution of thin-film multi-layer stacks, taking into account interference effects. The optical constants of the component materials (*n* and *k*) were measured by spectroscopic ellipsometry and verified by transmission measurements. The wavelength-dependent absorbance in a given layer is calculated as the difference of the energy density flowing into and out of the layer. The number of absorbed photons is then obtained by convolution of the absorbance and the AM1.5G solar spectrum. To calculate the J_{sc} values from the number of absorbed photons, we assumed an IQE of 65% for DPP:PC_{60}BM and 75% for PCDTBT:PC_{70}BM and OPV12:PC_{60}BM sub-cells which were measured in their corresponding single-junction devices. During the simulation, we fixed the thicknesses of PCDTBT:PC_{70}BM and OPV12:PC_{60}BM to 80 nm and 200 nm, respectively, which are the same with their optimized thickness in their single-junction cell with evaporated opaque electrode. For hybrid triple-junction cell, perovskite/DPP-DPP, the film thickness of the perovskite layer was fixed to 200 nm during the calculation of the current density distribution in the three subcells. We used a 100% IQE value for the perovskite which has been confirmed in our group[7] as well as in the literature[8].

Supplementary Methods

Semitransparent perovskite cells fabrication

$PbCl_2$ (99.98%, Alfa Aesar) and CH_3NH_3I (provided by Dyenamo) mixed with mole ratio of 1:3 were dissolved in dimethyl formamide in a concentration of 30%. The precursor solution was stirred at 60 ^{o}C for 3 hours inside a N_2-filled glovebox before use. Prior to spin coating, the solution was filtered using 0.2 μm PTFE syringe filter. $PC_{60}BM$ (99.5%, Solenne BV) was dissolved in chlorobenzene with a concentration of 20 mg/mL.

The patterned ITO-coated glass substrates were subsequently ultrasonic cleaned with acetone and isopropanol for 10 minutes each. On cleaned ITO substrate, a layer of PEDOT:PSS (Clevios^{TM} P VP AI 4083) was deposited by doctor blading at 50 ^{o}C and annealed at 140 ^{o}C for 10 minutes, giving a ~40 nm thick film. Afterwards, the substrates were transferred into the glovebox for the deposition of perovskite absorber, $PC_{60}BM$ and ZnO layers.

The filtered perovskite precursor was spin-cast at 2000 rpm for 45 seconds. The crystallization of the perovskite was performed by annealing the as-casted film from 50 ^{o}C to 110 ^{o}C with duration of 40 minutes, and subsequently kept at 110 °C for another 40 minutes. The thickness of $CH_3NH_3PbI_{3-x}Cl_x$ layer is approximately 200 nm. $PC_{60}BM$ solution was then spin-cast onto $CH_3NH_3PbI_{3-x}Cl_x$ layer at 1000 rpm for 45 seconds. Then, the ZnO solution was spin-cast onto $PC_{60}BM$ at a different speed of 2000 rpm for 45 seconds. To finalize the device fabrication, AgNWs diluted with isopropanol (1:3 vol%) was deposited onto ZnO layer at 45 ^{o}C under air atmosphere using a home-built spray coater.[9] The obtained 100-nm-thick AgNW film had a sheet resistance of ~20 ohm sq^{-1} and a transmittance of ~85% at 550 nm.

Supplementary References:

1. Guo, F. *et al.* Solution-processed parallel tandem polymer solar cells using silver nanowires as intermediate electrode. *Acs Nano* **8,** 12632-12640 (2014).
2. Scharber, M. C. *et al.* Design rules for donors in bulk-heterojunction solar cells - Towards 10 % energy-conversion efficiency. *Adv. Mater.* **18,** 789-794 (2006).
3. Dennler, G. *et al.* Design rules for donors in bulk-heterojunction tandem solar cells-towards 15 % energy-conversion efficiency. *Adv. Mater.* **20,** 579-583 (2008).
4. Veldman, D., Meskers, S. C. J. & Janssen, R. A. J. The Energy of Charge-Transfer States in Electron Donor-Acceptor Blends: Insight into the Energy Losses in Organic Solar Cells. *Adv. Funct. Mater.* **19,** 1939-1948 (2009).
5. Centurioni, E. Generalized matrix method for calculation of internal light energy flux in mixed coherent and incoherent multilayers. *Appl. Opt.* **44,** 7532-7539 (2005).
6. Pettersson, L. A. A., Roman, L. S. & Inganas, O. Modeling photocurrent action spectra of photovoltaic devices based on organic thin films. *J. Appl. Phys.* **86,** 487-496 (1999).
7. Guo, F. *et al.* High-performance semitransparent perovskite solar cells with solution-processed silver nanowires as top electrodes. *Nanoscale* **7,** 1642-1649 (2015).
8. Sun, S. Y. *et al.* The origin of high efficiency in low-temperature solution-processable bilayer organometal halide hybrid solar cells. *Energy Environ. Sci.* **7,** 399-407 (2014).
9. Guo, F. *et al.* ITO-Free and Fully Solution-Processed Semitransparent Organic Solar Cells with High Fill Factors. *Adv. Energy Mater.* **3,** 1062-1067 (2013).
